# Distinct nitrogen cycling and steep chemical gradients in *Trichodesmium* colonies

**DOI:** 10.1038/s41396-019-0514-9

**Published:** 2019-10-21

**Authors:** Isabell Klawonn, Meri J. Eichner, Samuel T. Wilson, Nasrollah Moradi, Bo Thamdrup, Steffen Kümmel, Matthias Gehre, Arzhang Khalili, Hans-Peter Grossart, David M. Karl, Helle Ploug

**Affiliations:** 1 Department of Ecology, Environment and Plant Sciences, Stockholm University, Stockholm, Sweden; 2 Department of Experimental Limnology, IGB-Leibniz-Institute of Freshwater Ecology and Inland Fisheries, Berlin, Germany; 3 Department of Marine Sciences, University of Gothenburg, Gothenburg, Sweden; 4 Centre A lgatech, Institute of Microbiology, The Czech Academy of Sciences, Trebon, Czech Republic; 5 Daniel K. Inouye Center for Microbial Oceanography, Research and Education, University of Hawai’i at Manoa, Honolulu, HI USA; 6 Department of Physics & Earth Sciences, Jacobs University Bremen, Bremen, Germany; 7 Max Planck Institute for Marine Microbiology, Bremen, Germany; 8 Department of Biology and Nordic Center for Earth Evolution, University of Southern Denmark, Odense M, Denmark; 9 Department of Isotope Biogeochemistry, Helmholtz Centre for Environmental Research (UFZ), Leipzig, Germany; 10 Present Address: Department of Earth System Science, Stanford University, Stanford, CA USA

**Keywords:** Biogeochemistry, Water microbiology

## Abstract

*Trichodesmium* is an important dinitrogen (N_2_)-fixing cyanobacterium in marine ecosystems. Recent nucleic acid analyses indicate that *Trichodesmium* colonies with their diverse epibionts support various nitrogen (N) transformations beyond N_2_ fixation. However, rates of these transformations and concentration gradients of N compounds in *Trichodesmium* colonies remain largely unresolved. We combined isotope-tracer incubations, micro-profiling and numeric modelling to explore carbon fixation, N cycling processes as well as oxygen, ammonium and nitrate concentration gradients in individual field-sampled *Trichodesmium* colonies. Colonies were net-autotrophic, with carbon and N_2_ fixation occurring mostly during the day. Ten percent of the fixed N was released as ammonium after 12-h incubations. Nitrification was not detectable but nitrate consumption was high when nitrate was added. The consumed nitrate was partly reduced to ammonium, while denitrification was insignificant. Thus, the potential N transformation network was characterised by fixed N gain and recycling processes rather than denitrification. Oxygen concentrations within colonies were ~60–200% air-saturation. Moreover, our modelling predicted steep concentration gradients, with up to 6-fold higher ammonium concentrations, and nitrate depletion in the colony centre compared to the ambient seawater. These gradients created a chemically heterogeneous microenvironment, presumably facilitating diverse microbial metabolisms in millimetre-sized *Trichodesmium* colonies.

## Introduction

*Trichodesmium* has a ubiquitous distribution throughout tropical and subtropical oceans where it contributes substantial amounts of new nitrogen (N) to the oligotrophic near-surface ocean through dinitrogen (N_2_) fixation [1, 2]. In the North Pacific Subtropical Gyre, *Trichodesmium* is suggested to account for up to half of the biologically fixed N_2_ [3], which generally mitigates N limitation, and even promotes the growth of a broader plankton community [4–6] and eventual carbon (C) export to the deep sea [7–9]. *Trichodesmium* grows as filaments, referred to as trichomes, which at times aggregate as millimetre-sized spindle-shaped (tufts) or spherical (puffs) colonies [10], often forming conspicuous blooms which can be observed from space [11]. These colonies have long been recognised as hot spots for N_2_ fixation [12, 13] but are increasingly suspected to host additional processes including N recycling and loss processes, as inferred from nucleic acid analyses [14–16].

The N cycle involves a series of oxidation and reduction processes [17]. Bioavailable N is gained through the conversion of N_2_ to ammonium which, in addition to regenerated ammonium, can be oxidised to nitrite and nitrate (nitrification) or incorporated into biomass (assimilation). In turn, particulate organic N can be recycled to ammonium (ammonification), while nitrate and nitrite can be reduced to ammonium via assimilatory and dissimilatory pathways, or transformed to N_2_ (denitrification). Additional complexity to the N cycle is added by dissolved organic nitrogen (DON), which comprises a heterogeneous mixture of N compounds of different lability [18]. The co-occurrence of aerobic and anaerobic N transformation pathways is typically observed at redoxclines, such as oxic–anoxic interfaces at sediment surfaces and in mesopelagic oxygen minimum zones [19, 20]. Nonetheless, aerobic and anaerobic N transformations have also been found in marine snow or cyanobacterial colonies in oxic waters [21, 22]. The latter potentially include *Trichodesmium* colonies, representing microenvironments distinctly different from the surrounding water in terms of their physico-chemical properties, metabolic functions and phylogenetic composition. For example, oxygen concentrations can decrease from 200% down to 10% air-saturation, and the pH from 8.8 down to 7.5, during a light–dark shift in *Trichodesmium* colonies [23–25]. Moreover, activities of enzymes, such as peptidases, are high in *Trichodesmium* colonies [26], and newly fixed N can get released as ammonium or dissolved organic N (e.g., amino acids) into the ambient water [27–29]. This release is suggested to lead to a region of elevated nutrient concentrations in close proximity to the colonies [30], referred to as phycosphere [31] or trichosphere. As a result, *Trichodesmium* colonies are favourable microhabitats for numerous epibionts in otherwise N depleted waters [15, 32, 33].

The association of epibionts with *Trichodesmium* has been recognised for several decades [34–37]. However, their taxonomic diversity and metabolic potential has only recently been unveiled by research into their nucleic acids [15, 16, 32, 33, 38–41]. Metagenomic studies showed that epibionts substantially expand the metabolic functionality in colonies compared to single trichomes [38]. With respect to N pathways, transcripts encoding for ammonium, nitrite and nitrate transporters, and for genes involved in assimilatory/dissimilatory nitrate reduction to ammonium and denitrification have been detected [14–16]. Thus, the metabolic potential of the *Trichodesmium* holobiont stretches beyond N_2_ fixation, suggesting an intimate spatial coupling of various N cycling processes. However, this metabolic potential is yet to be confirmed by actual rate measurements.

Here, we complement the previously observed metabolic potential in the *Trichodesmium* holobiont with rate measurements of co-occurring N transformation processes. We used stable-isotope incubations to quantify N gain, recycling and loss processes in field-sampled *Trichodesmium* colonies. For simplicity, we focused on processes transforming inorganic N speciesc, like N_2_ fixation, ammonium release, nitrification, denitrification and nitrate reduction to ammonium/nitrite, without including dissolved organic N speciesc or assimilation processes other than C/N_2_ fixation in our analyses. Moreover, we measured oxygen profiles in individual colonies, and modelled microscale gradients of oxygen, ammonium and nitrate in the trichosphere, to characterise the chemical microenvironment in *Trichodesmium* colonies. Our quantitative estimates reveal the complexity of N cycling processes and microscale heterogeneity in *Trichodesmium* colonies.

## Materials and methods

### Sampling and environmental data

Seawater and puff-shaped *Trichodesmium* colonies were sampled at Station ALOHA (22°45′N 158°00′W) in the oligotrophic North Pacific Subtropical Gyre, in the frame of the Hawai’i Ocean Time-series program (HOT cruise #265), during September 2014. Colonies were collected from 0 to 10 m water depth using a plankton net (200 μm mesh-size, Aquatic Research Instruments). Seawater temperature was 27 °C and salinity 34.4. Further biogeochemical properties of the surface water are listed in Supplementary Table S1. We alert the reader that our experiments were conducted in parallel to a separate study on *Trichodesmium* under varying partial pressures of carbon dioxide [23]. We therefore refer to this previously published study when appropriate for shared analytical methods and complementary datasets, such as colony characteristics and single-cell activities.

### Colony characteristics

Colony characteristics, including chlorophyll *a* content, *Trichodesmium* species composition and epibionts, cell numbers and dimensions, and particulate organic carbon and nitrogen contents (POC and PON) were analysed as presented in Eichner et al. [23]. In brief, chlorophyll *a* was analysed fluorometrically after extraction in 90% acetone. *Trichodesmium* species were tentatively identified from Lugol-preserved colonies via microscopy based on cell shape and size [42]. Heterotrophic bacteria and further epibionts were examined on colonies filtered onto polycarbonate filters using an epifluorescence microscope and scanning electron microscope. POC and PON contents, and POC:PON ratios were determined from colonies filtered onto precombusted GF/F filters, and analysed using elemental analysis isotope-ratio mass spectrometry (EA-IRMS, see below).

### Stable-isotope incubations

Stable-isotope incubations were conducted during day-time (6AM–6PM) and night-time (6PM–6AM), with colonies being collected at 5AM (September 16) and 5PM (September 14), respectively. Colonies were individually transferred with an inoculation loop into 0.2 µm filtered water (to remove loosely associated biota), and thereafter into 5.9 ml Exetainer vials (Labco, Lampeter, UK) with 0.2 µm filtered surface seawater, adding five colonies per vial. Isotopically labelled substrates were added to measure C fixation and specific N transformation processes (Table 1). Incubation #1 was enriched with pre-dissolved ^13^C-dissolved inorganic carbon (NaH^13^CO_3_, Sigma-Aldrich) and ^15^N_2_ (Cambridge Isotope Laboratories), to quantify C fixation, N_2_ fixation and ammonium release during active N_2_ fixation. ^15^N_2_ was added as an aliquot of ^15^N_2_-enriched seawater, which was prepared from 0.2 µm filtered seawater following [43]. An additional five replicates of incubation #1, with only one colony per vial, were used to determine N_2_ and C fixation rates in single *Trichodesmium* cells using secondary-ion mass spectrometry (SIMS), as presented previously [23]. The ^13^C-labelling was 4.2 ± 0.2% (mean ± s.d., *n* = 6, quantified by trace gas IRMS, UC Davis California, USA, precision ± 0.1‰) and the ^15^N-labelling was 4.2 ± 0.4% (mean ± s.d., *n* = 7, quantified by membrane inlet mass spectrometry, precision ± 0.1‰). Incubations #2–6 were enriched with ^15^N-nitrate, ^15^N-nitrite or ^15^N-ammonium (Na^15^NO_3_, Na^15^NO_2_ and ^15^NH_4_Cl, ^15^N ≥ 98 atom%, Sigma-Aldrich) to final concentrations of 0.9 ± 0.3 µM (mean ± s.d., *n* = 18), equal to ^15^N-labelling of 55, 94 and 79% in the substrate pool, respectively. These final concentrations exceeded typical in situ concentrations, in order to overcome diffusion-limited solute transport into the colonies (see supplementary text S1 for further explanations). Concentrations of dissolved inorganic nitrogen (DIN: ammonium, nitrate, nitrite) and ^15^N-additions were determined from incubations #6 and #7. The water was gently 0.45 µm-filtered (cellulose–acetate, Sartorius) into acid-washed Falcon tubes and stored at −20 °C until nutrient analyses following [44] at the College of Earth, Ocean and Atmospheric Sciences, Oregon State University. The detection limit was 50 nmol L^−1^ (precision/accuracy ± 1%). Incubation #5 was enriched with 86 µM *N*-Allylthiourea (ATU, Sigma-Aldrich) in addition to ^15^N-nitrate, to inhibit the ammonium oxidation step of nitrification [45] without any inhibitory effect on denitrification or anammox [46].

**Table 1:** Additions of ^15^N- and ^13^C-labelled compounds during isotope-tracer incubations, to distinguish C fixation and specific N pathways within *Trichodesmium* colonies

Incubation #	Added substrate (isotopically labelled)	Targeted pathway	Targeted product (isotopically labelled)
(1)	^15^N_2_, ^13^C-DIC	N_2_ fixation and C fixation (single-colony and single-cell level), ammonium release	PO^15^N, PO^13^C, ^15^NH_4_^+^
(2)	^15^NH_4_^+^	Nitrification	^15^N_2_O, ^15^NO_3_^−^, ^15^NO_2_^−^
(3)	^15^NO_2_^−^	Nitrification: NO_2_^−^ reduction to NH_4_^+^/N_2_O/N_2_	^15^NH_4_^+^, ^15^N_2_O, ^15^N_2_
(4)	^15^NO_3_^−^	NO_3_^−^ reduction to NH_4_^+^/NO_2_^−^, denitrification, total NO_3_^−^ consumption	^15^N_2_, ^15^N_2_O, ^15^NH_4_^+^, ^15^NO_2_^−^, ^15^NO_3_^−^
(5)	^15^NO_3_^−^ plus ATU (nitrification inhibitor)	NO_3_^−^ reduction to NH_4_^+^/NO_2_^−^, denitrification, total NO_3_^−^ consumption	^15^N_2_, ^15^N_2_O, ^15^NH_4_^+^, ^15^NO_2_^−^, ^15^NO_3_^−^
(6)	^15^NH_4_^+^, ^15^NO_2_^−^ or ^15^NO_3_^−^	Control, isotope labelling %	^15^NO_3_^−^, ^15^NO_2_^−^, ^15^NH_4_^+^, total NO_3_^−^, NO_2_^−^, NH_4_^+^
(7)	No additions	Net changes in NO_3_^−^, NO_2_^−^, NH_4_^+^ isotope labelling %	total NO_3_^−^, NO_2_^−^, NH_4_^+^

*PO*^*15*^*N* particulate organic ^15^N-nitrogen, *PO*^*13*^*C* particulate organic ^13^C-carbon, *ATU* N-Allylthiourea

Five replicate vials with isotope additions and colonies, and five control vials with isotope additions but without colonies were set up for each incubation (#1–6). No isotopes were added to incubation #7. The Exetainers were closed headspace-free and placed in an incubator on deck, cooled with flowing surface water and shaded to 50% surface irradiance (blue acrylic shielding #2069 Delvie’s Plastic Inc., USA). The Exetainers were attached horizontally onto thin wires in the water, to allow for gentle movements during water flow and ship movement, thus keeping colonies in suspension and decreasing diffusion-limited solute transport to the colonies, as compared to colonies that would settle onto the vials’ bottom [47]. Incubations were terminated by injecting 0.05 mL saturated ZnCl_2_ or HgCl_2_ to each Exetainer. HgCl_2_ was added to vials for later ^15^N-nitrate analyses, to avoid a strong lowering in pH as caused by ZnCl_2_.

Twenty-five colonies (5 colonies × 5 replicates) from incubation #1 were pooled per GF/F filter (25 mm, Whatman). Filters were dried at 50 °C overnight, fumed over HCl, pelletized into tin cups and analysed by EA-IRMS (UC Davis, precision ± 0.2‰ for ^13^C and ±0.3‰ for ^15^N, using Vienna PeeDee Belemnite and air as C and N standards, respectively). Rates of N_2_ and C fixation were calculated following [48]. The filtrate was stored in the Exetainer vials for later analysis of released ^15^N-ammonium.

The production of ^15^N-labelled N_2_, N_2_O, nitrate, nitrite and ammonium was determined by headspace analysis using gas chromatography IRMS (GC-IRMS, concentration precision ± 5%) at the University of Southern Denmark in Odense and the UFZ in Leipzig, Germany. Both GC-IRMS set-ups and the analytical procedure are specified in supplementary text S2. Production of N_2_, N_2_O, ammonium, nitrate and nitrite was calculated from the ^15^N-excess concentrations relative to air, and corrected for the ^15^N mol fraction in the N pool in control samples. Rates were calculated from the production of each N compound versus time per colony, and tested against controls for statistical significance (*t* test at a confidence interval of 95% for normally distributed variables; Mann–Whitney *U*-test for non-normally distributed variables). Rates not significantly different from controls were defined as not detectable. DIN concentrations were substantially enhanced by 55–94% after ^15^N-isotope additions, potentially stimulating (or inhibiting) N transformation processes in strictly N limiting water. Thus, all rates (except for N_2_/C fixation and ammonium release) should be considered as potential rates, and moreover as net rates due to concurrent production and consumption processes.

### Oxygen microsensor analyses

Oxygen concentration measurements were done under light and dark conditions (at 1000 and 0 μmol photons m^−2^ s^−1^, respectively), as described in [23]. Single colonies were placed in a laminar flow (0.1 mm s^−1^, similar to natural floating or sinking velocities) [49] of filtered aerated seawater in a temperature-controlled (25 °C) flow-through chamber [50]. Oxygen concentrations were measured with a Clark-type oxygen microelectrode (10 µm, Unisense, Denmark) at a vertical resolution of 100 µm from the ambient water towards the colony centre [51]. Oxygen fluxes *J* were calculated according to Fick’s first law (supplementary eq. S1) [50], applying a diffusion coefficient *D* of 2.24 × 10^−5^ cm^2^ s^−1^ (25 °C, salinity 34). Fluxes were normalised to entire colonies using the colony surface areas, calculated from the colony radius assuming spherical geometry.

### Numerical modelling: concentrations of oxygen, ammonium and nitrate

Concentration profiles and distribution fields of oxygen, ammonium and nitrate were simulated for colonies and single trichomes, using a recently developed advection–diffusion–reaction model [52]. This model is applicable to simulate small-scale fluxes of gases and nutrients in porous phytoplankton colonies, whose chemical microenvironments are driven by diffusive and advective mass transfer, as well as by metabolic activities.

*Trichodesmium* colonies have a complex geometry, and are able to ascend, descend or remain neutrally buoyant in the water column [49]. Simulating realistic flow fields around/inside *Trichodesmium* colonies is therefore challenging and computationally expensive. Moreover, interstitial voids in highly porous phytoplankton colonies and aggregates are filled with viscous polymers [53] which inhibit advective flow [54]. For simplicity, we thus neglected flow (advection) effects on the concentration field around/inside colonies and single trichomes, with the main goal of estimating concentration profiles from the centre to the ambient water at still water conditions.

**Fig. 1:**
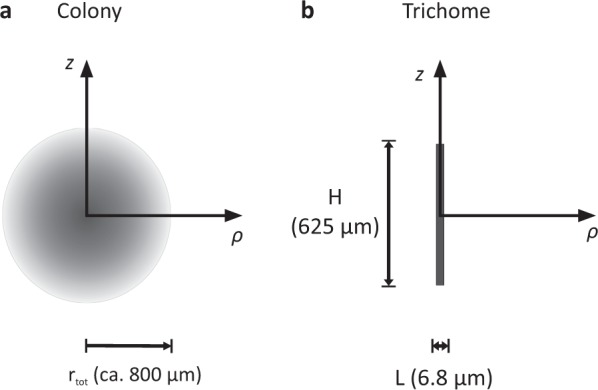
Schematic cross-section of the modelled colony as a porous sphere with variable porosity (**a**) and single trichome as a solid cylinder (**b**), both with geometrical symmetry along the *z*-axis. Cells were considered as solid objects. In the colony, a radially decreasing cell density towards the colony surface was assumed, as illustrated by the gradual shading (**a**). The distance from the colony centre *r*, as used in Eq. 4, was defined as ${{r = }}\sqrt {\rho ^2{\mathrm{ + }}z^2}$

Colonies were modelled as porous spheres, and trichomes as non-porous solid cylinders (Fig. 1). The diffusion–reaction equation

1 $$\varepsilon \frac{{{\mathrm{d}}C_\alpha }}{{{{{\mathrm{d}}t}}}} = \nabla {\mathrm{.}}\left( {\varepsilon D_\alpha \nabla C_\alpha } \right){\mathrm{ + }}nR_\alpha.$$

was solved numerically to calculate the (extracellular) concentration of the solute *C*_*α*_ (*α* specifies the considered solute) within the computational domain, with *t* as time, ∇ as gradient operator, *D*_*α*_ as diffusion coefficient, *ε* as local porosity and *n* as local number of cells in the representative elementary volume. The reaction term *R*_*α*_ denotes the solute consumption/release rate by each cell in the colony, accounting for Michaelis–Menten kinetics. *R*_*α*_ for oxygen and nitrate consumption were modelled according to the first-order kinetics

2 $$R_\alpha = \frac{{V_{{{{\mathrm{m}}(\alpha )}}} \times {{C}}_\alpha }}{{K_{{\mathrm{m{(\alpha )}}}} + {{C}}_\alpha }},$$

where *K*_m(*α*)_ represents the half-saturation coefficient, and *V*_m(*α*)_ the maximum reaction rate for each solute. *R*_*α*_ for oxygen and ammonium release were modelled according to zeroth-order kinetics

3 $$R_\alpha = V_{{\mathrm{m}}\left( \alpha \right)}$$

with *V*_m(*α*)_ as constant (maximum) reaction rate. *K*_m(*α*)_ for oxygen respiration was set to 1 µM [55], and for nitrate reduction and consumption to 20 nM. The latter has been measured for nitrate assimilation of natural plankton communities under N depletion [56], and seems justified given that assimilation was presumably the dominating nitrate consumption pathway in our incubations (see Discussion). *V*_m(*α*)_ derived from the colony-specific activity rates, measured after adding artificially high substrate concentration of ~1 µM (see above) during stable-isotope incubations, normalised to single-cell units (Table S2). Outside the colony and trichome, the reaction rate was set to *R*_*α*_ = 0 and the porosity to *ε* = 1. For all simulations, temperature was 25 °C and salinity 34.

In the modelled colony, we considered a decreasing cell density along the radius. The volume fraction *v* that was occupied by cells within a representative elementary volume inside the colony equalled *v* *=* 1 − *ε*. At a certain radius *r*, it was calculated as

4 $$v{{ = {b} \times }}\left[ {{\mathrm{1 + tanh}}\left( {{\mathrm{2}} - \left( {{{2r/}}r_{{\mathrm{tot}}} - {\mathrm{0}}{\mathrm{.6}}} \right){\mathrm{/0}}{\mathrm{.3}}} \right)} \right],$$

where *r*_tot_ is the total radius and *b* = 1.7141 × 10^−3^. The parameter *b* was determined using the condition that the integral of *v* must equal the total volume of cells in one colony. Equation 4 was derived by trial and error, with the aim to distribute the cells within the colony in a plausible way. The plausibility was based on the measured number of cells per colony, the cell dimensions (see below) and the colony volume, as well as on photographs of *Trichodesmium* colonies (see supplementary Fig. S1), which suggested higher cell densities in the colony centre, and a radial decrease towards zero at the colony surface. The local number of cells was *n* *=* *v* *×* Ω*/v*_cell_, with Ω as the actual volume of the elementary volume, and *v*_cell_ as average volume of individual cells.

To solve Eq. 1 numerically in the computational domain, we used the lattice Boltzmann method [52, 57]. A detailed description of this method, including underlying assumptions, boundary conditions and input parameters, is included in the supplementary material (Text S3, Fig. S2, and Table S2). The model code can be requested directly from the authors (N.M., nmoradi@marum.de).

## Results

### Colony characteristics

*Trichodesmium* was tentatively identified as *T. thiebautii*, *T. erythraeum* and *T. tenue* (Table S3 in ref. [23]) at proportions of 60%, 30% and 10%, respectively. The average cell size was 8.8 × 6.8 µm (length × width, Table 2), as estimated from the proportions of species and cells sizes. Cell abundances were ~6000 cells colony^−1^. Epibionts consisted predominantely of heterotrophic bacteria (4–5 per *Trichodesmium* cell), whereas picocyanobacteria, dinoflagellates and fungi were rare, mostly limited to one individual of each per colony. Further characteristics of colonies and cells are listed in Table 2.

**Table 2:** Characteristics of *Trichodesmium* colonies and cells

Characteristic (unit)	Value (no. of replicates)
POC (nmol C colony^−1^)	248 ± 51 (*n* = 23)
PON (nmol N colony^−1^)	39 ± 10 (*n* = 23)
POC:PON ratio	6.5 ± 0.6 (*n* = 23)
Chlorophyll *a* (ng chl *a* colony^−1^)	14 ± 4 (*n* = 5)^a^
Cells per colony	5946 ± 6852 (*n* = 22)^a^
Colony radius (mm)	0.5–1.0 (*n* = 14)
Cell dimensions/volume (µm, length × width/µm^3^)	8.8 × 6.8 (range: 4–15 × 4–14)/317

^a^Values derived from Table 1 in Eichner et al. [23]

### Nitrogen transformation processes and C fixation (stable-isotope incubations)

Colony-specific C and N_2_ fixation occurred mostly at day-time, and less at night-time (Table 3). The same diel pattern was observed for single *Trichodesmium* cells analysed by SIMS (for details see [23]). For these, C growth rates based on C fixation were on average 0.20 d^−1^, and N growth rates based on N_2_ fixation 0.03 d^−1^ during the day but negligible at night-time (Table 3). Ammonium release during N_2_ fixation was only detectable during day-time, and equalled approximately 10% of net N_2_ fixation (Table 3).

**Table 3:** Net rates of C fixation, N cycling pathways and oxygen production/respiration in *Trichodesmium* colonies. C and N_2_ fixation were additionally analysed for single cells

Incubation #	Pathway	Net rate (pmol colony^−1^ h^−1^)		Replicates
	Substrate → product	Day (6AM–6PM)	Night (6PM–6AM)	
**Isotope-tracer incubations**				
	*C fixation*			
(1)	DIC → POC	2097.6	66.4	25 colonies pooled/5 vials
(1)	DIC → Single-cell C fixation	0.20 ± 0.03 (d^−1^)^a^	0.0012 ± 0.0006 (d^−1^)^a^	Day: 117 cells Night: 51 cells
	*N*_2_ *fixation*			
(1)	N_2_ → PON	212.3	0.9	25 colonies pooled/5 vials
(1)	N_2_ → Single-cell N_2_ fixation	0.03 ± 0.02 (d^−1^)^a^	0.0004 ± 0.0002 (d^−1^)^a^	Day: 117 cells Night: 51 cells
	*Ammonium release during N*_2_ *fixation*			
(1)	N_2_ → NH_4_^+^	19.4 ± 14.0 (7.3–41.2)^b^	N/D	5
	*Nitrification*			
(2)	NH_4_^+^ → NO_2_^−^	N/D	N/D	5
(2)	NH_4_^+^ → NO_3_^−^	N/D	N/D	5
	*Nitrate/nitrite reduction*			
(4)	NO_3_^−^ → NO_2_^−^	5.7 ± 4.1 (3.5–13.1)	4.8 ± 4.0 (1.9–11.9)	5
(4)	NO_3_^−^ → NH_4_^+^	5.0 ± 4.5 (0.9–12.3)	10.9 ± 7.9 (2.4–20.0)^b^	5
(3)	NO_2_^−^ → NH_4_^+^	1.6 ± 0.8 (0.5–2.5)	1.1 ± 0.3 (0.6–1.4)	5
	*Residual nitrate consumption*			
(4)	Total NO_3_^−^ decrease minus NO_3_^−^ reduction to NH_4_^+^/N_2_	65.7 ± 46.2 (4.3–122.1)	67.8 ± 50.9 (24.3–153.5)^b^	5
	*Denitrification*			
(4)	NO_3_^−^ → N_2_	N/D	N/D	5
(3)	NO_2_^−^ → N_2_	N/D	N/D	5
	*N*_2_*O production*			
(4)	NO_3_^−^ → N_2_O	N/D	N/D	5
(5)	NO_3_^−^ (+ATU) → N_2_O	0.52 ± 0.14 (0.32–0.64)	N/D	5
(3)	NO_2_^−^ → N_2_O	N/D	N/D	5
(2)	NH_4_^+^ → N_2_O (nitrification)	0.02 ± 0.01 (0.01–0.04)	N/D	5
**Microsensor analyses**				
		Light	Dark	
	Oxygen production	2868 ± 2333 (−1752 to 6849)^b^	–	21 (profiles)
	Oxygen respiration	–	1751 ± 1360 (529–5282)^b^	11 (profiles)

Presented rates were significantly different from control samples (*p* < 0.05), whereas nonsignificant rates are denoted as N/D (not detectable). The substrate and product indicate the added and measured isotopically labelled compounds, respectively, during isotope-tracer incubations. Listed are averages ± stdev with ranges in brackets.

^a^Note the different unit for single-cell rates. Rates derived from Table 3 in Eichner et al. [23] and were corrected for controls (cells incubated without isotope additions)

^b^Rates used to calculate maximum reaction rates *V*_m_ for model simulations (average rate divided by 6000 cells, see Table S2)

**Fig. 2:**
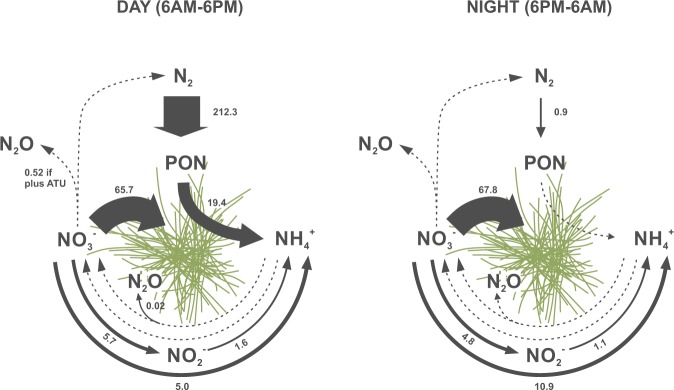
Conceptual overview of the potential N transformation network in *Trichodesmium* colonies, based on stable-isotope incubations during day- and night-time. Solid arrows indicate processes with significant rates (pmol N h^−1^ colony^−1^). Dashed arrows indicate those with insignificant (non-detectable) rates. The arrow widths present an approximation for the magnitude of each transformation. For comparison, rates of the displayed processes are listed in Table 3. ATU *N*-Allylthiourea, PON particulate organic nitrogen

Significant rates of nitrification were not detected. Yet, the potential nitrate consumption was high (Table 3, Fig. 2). We distinguished between (i) nitrate reduction to nitrite and ammonium, (ii) complete denitrification, i.e., nitrate loss as N_2_, and (iii) residual nitrate consumption specified as total decrease of added ^15^N-nitrate minus (i) and (ii). Approximately 10% of the consumed nitrate was reduced to ammonium and/or nitrite after 12-h incubations during day- and night-time. Complete denitrification was insignificant. Rates of nitrite reduction to ammonium were significantly lower than those for nitrate reduction to nitrite or ammonium (*p* < 0.05, *t* test). N_2_ and N_2_O production were not detected in most cases after ^15^N-nitrate, nitrite or ammonium additions, except for (i) low N_2_O production (mass 29) in ^15^N-ammonium incubations and (ii) N_2_O production (mass 30) after ^15^N-nitrate incubations in combination with *N*-Allylthiourea an inhibitor for nitrification, both during day-time (Table 3).

#### Control measurements

Bulk concentrations of ammonium, nitrate and nitrite in the control vials (incubation #7, with and without colonies) were 0.19 ± 0.15, 0.40 ± 0.03 µmol L^−1^ and 0.05 ± 0.02 µmol L^−1^ (*n* = 4), respectively, at time zero and did not change significantly after 12-h incubations (*t* test, *p* > 0.05).

### Oxygen microsensor analyses

**Fig. 3:**
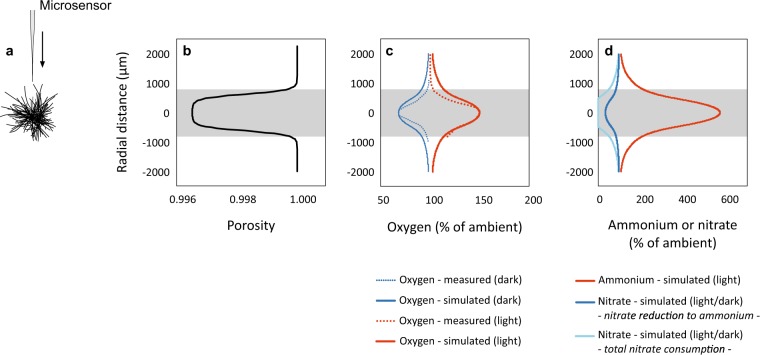
**a** Oxygen profiles were measured along the central *z*-axis of single *Trichodesmium* colonies. **b** The best model fit was obtained for the shown porosity, estimated from cell distributions along the colony radius (*ε* = 1 − *v*, Eq. 4). The porosity shows almost constant (slightly increasing) values in the core of the colony, and then increases towards one at the colony surface. The integrated number of cells within the entire colony volume resulted in the total number of cells (~6000 cells colony^−1^, with an average cell volume of 317 µm^3^, based on the average cell dimensions listed in Table 2). Note, the porosity was based on the volume occupied by *Trichodesmium* cells only, excluding other similar sized organisms that were hardly present or viscous polymers. **c** Direct comparison of measured and simulated oxygen profiles. **d** Simulated ammonium profiles to support the measured ammonium release (during N_2_ fixation at day-time), and nitrate profile to support the measured rates of nitrate reduction to ammonium and overall consumption (averaged for day- and night-time). The grey-shaded areas highlight the colony area (*r* ~ 800 µm)

We recorded 32 oxygen profiles (21× light/11× dark), showing steep oxygen gradients from the ambient water towards the colony centre (Fig. 3). Oxygen fluxes *J* were 0.039 ± 0.032 nmol cm^−2^ s^−1^ (range: −0.024 to 0.093, *n* = 21) during light due to net photosynthesis, and −0.024 ± 0.019 nmol cm^−2^ s^−1^ (range: −0.007 to −0.072, *n* = 11) during darkness due to respiration. These fluxes lead to supersaturation of 145 ± 36% (92–203%) and undersaturation of 78 ± 12% (56–98%) in the colony centre, respectively. Rates of oxygen production exceeded those of respiration (Table 3), demonstrating that colonies were net autotrophic. Complementary oxygen data (at different *p*CO_2_ and day-times) during the same field campaign are available in [23].

### Numerical modelling: concentrations of oxygen, ammonium and nitrate

The model output was validated against oxygen profiles measured for one *Trichodesmium* colony, whose size (radius ~ 800 µm), morphology (puff-shape) and oxygen profiles were representative for the investigated colonies. Applying the shown porosity (Fig. 3b), the model could successfully reproduce the measured oxygen concentrations of 150 and 68% air-saturation in the colony centre during light and darkness, respectively (Fig. 3c). The curve shapes were also similar, resulting in comparable oxygen fluxes at the colony–water interface (*J*_measured_ = 0.044 vs. *J*_modelled_ = 0.035 nmol cm^−2^ s^−1^ during light, and *J*_measured_ = −0.028 vs. *J*_modelled_ = −0.023 nmol cm^−2^ s^−1^ during darkness).

**Fig. 4:**
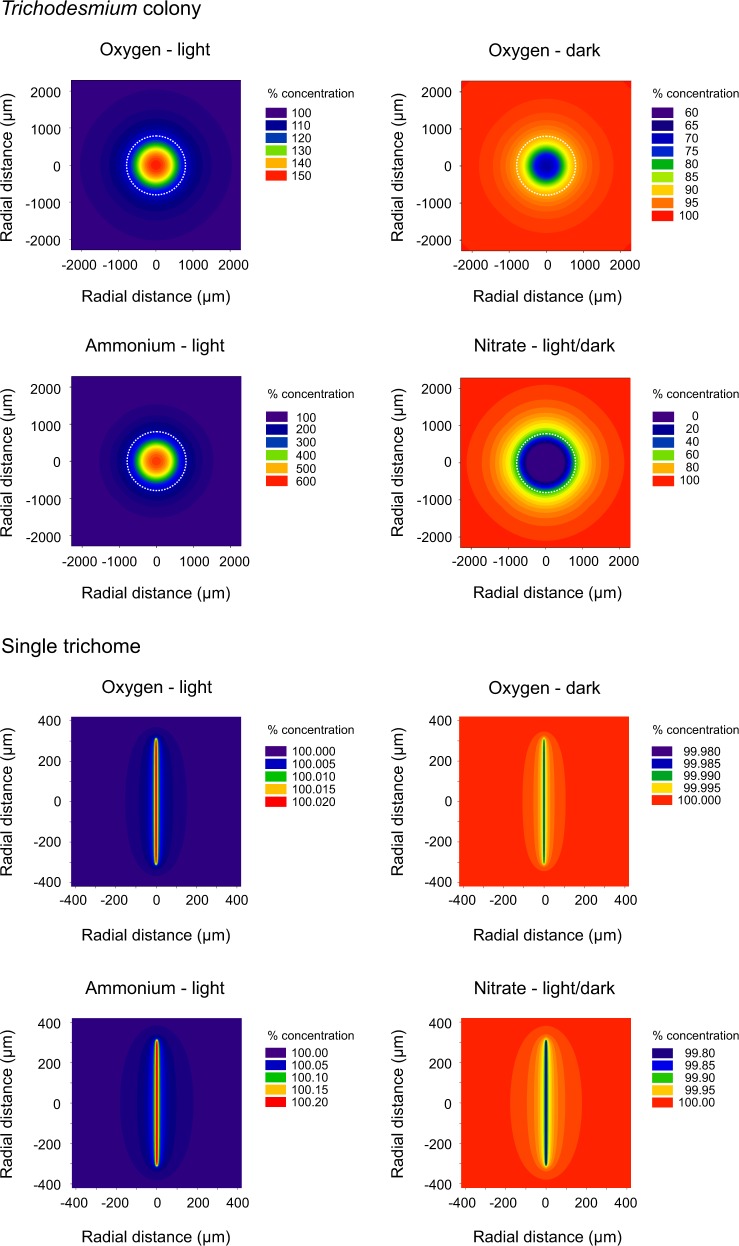
Concentration fields (in % of ambient concentration) simulated for *Trichodesmium* colonies and free trichomes. The colony outline is indicated as dashed circle in the four upper graphs. *x*- and y-axes represent *ρ* and *z*, respectively, as shown in Fig. 1. Note the different colour scales for each image

In addition to oxygen profiles, we modelled those for ammonium and nitrate. During day-time, newly fixed N was partly released as ammonium. Accordingly, the model predicted a steep ammonium concentration gradient, with ammonium concentrations being elevated to 570% (1.1 µM) in the colony centre compared to the ambient water (0.2 µM) (Figs. 3d and 4). Based on the measured rates of nitrate reduction to ammonium during day- and night-time, the model predicted nitrate depletion down to 34% (0.3 µM) in the colony centre relative to the ambient water (1.0 µM). Total nitrate consumption rates even resulted in nitrate depletion down to zero in the colony centre (Figs. 3d and 4). The nitrate-depleted core corresponded to approximately half of the colony radius, or 17% of its volume. In free trichomes, concentrations of ammonium, nitrate and oxygen were enriched/depleted by less than 1% compared to the ambient (Fig. 4). The data plotted in Figure 3 and 4 are archived in the PANGAEA database (https://www.pangaea.de/).

## Discussion

### Microscale heterogeneity in the trichosphere

*Trichodesmium* colonies represent highly heterogeneous microenvironments with dynamic physical, chemical and biological conditions, that change over micrometres [23–25] and within minutes [58]. Steep nutrient gradients in *Trichodesmium* colonies have often been proposed but rarely measured, since microsensors for relevant N compounds are not available for measurements in seawater. We used stable-isotope incubations in combination with microsensor measurements and computational simulations, to reveal the microscale growth conditions and N transformation processes inside N_2_-fixing *Trichodesmium* colonies. Our simulations predicted that ammonium concentrations were almost 6-fold enriched in the colony centre, owing to ammonium release from N_2_ fixation. Importantly, this enrichment might have been lower during our incubations due to a rapid ammonium turnover within colonies, as discussed below. It may, however, also increase due to enhanced ammonium production during organic matter remineralisation in decaying colonies, microbial infections or zooplankton grazing [18]. For instance, in colonies of N_2_-fixing cyanobacteria in the Baltic Sea, ammonium concentrations were predicted to be up to 60-fold enriched within their centre compared to the ambient water [22, 59, 60].

The trichosphere was 4–13-fold larger in its volume when compared to the colony volume itself (Fig. 4). The trichosphere was defined as the region that was enriched/depleted in nutrients and gases by at least 2% in the immediate surrounding of the colonies compared to the ambient water. Such diffusing nutrient patches of, e.g., ammonium, are likely to attract motile chemotactic microbes [61], and thus *Trichodesmium* colonies may become more heavily colonised by epibionts than free trichomes. In support of this hypothesis, typical epibionts on *Trichodesmium* colonies include Bacteroidetes and Gammaproteobacteria (e.g., *Alteromonas*, *Pseudoalteromonas*) [15, 32, 38, 40], which are recognised as fast-growing motile bacteria that utilise labile material, and may accelerate the N and C turnover in the colonies. The boundary layers that we derived from our simulations at still water conditions were 500–1000 µm, thus stretching over more than one radius, consistent with previously measured oxygen profiles [35]. Yet, in nature, shear forces at the colony surface due to turbulence are expected to decrease the boundary layer thickness. Exemplary, at common shear rates in surface waters (0.1–1.0 s^−1^), the effective boundary layer thickness would decrease down to ~200–500 µm (for calculations see [62]).

### C fixation, N_2_ fixation, ammonium release and N assimilation

Primary production and N_2_ fixation in *Trichodesmium* colonies were largely restricted to daylight hours, supporting specific growth rates of 0.10 and 0.02 d^−1^, respectively (considering a 12-h light/12-h dark period for rates given in Table 3), as common for *Trichodesmium* grown in nature [63–66]. About 10% of the newly fixed N was recovered as ammonium in the ambient water. Similar release rates of ammonium have been reported for natural populations in the South Pacific [4, 67]. Consistently, concentrations of ammonium (and DON) have been shown to be enriched in surface waters during *Trichodesmium* blooms compared to pre-bloom conditions at Station ALOHA [68].

Intriguingly, ambient ammonium concentrations remained at steady-state during our 12-h incubations, despite the ammonium supply from N_2_ fixation, and potential nitrate reduction to ammonium. The steady-state concentrations indicated a fast ammonium turnover within *Trichodesmium* consortia [64], as generally common in N depleted water [69, 70]. Possibly, ammonium was rapidly assimilated for growth. In single cells of *Trichodesmium*, C growth rates based on C fixation were six times as fast as their N growth rates based on N_2_ fixation (Table 3). Similarly, the ratio of colony-specific C to N_2_ fixation over one diel cycle was 10, in agreement with previously reported ratios of 9–67 [64]. The ratio of 10 indicated a N deficit of 60 pmol colony^−1^ h^−1^, given that the POC:PON ratio of colonies was 6.5 (Table 2). This deficit was moderated by 16% through ammonium release during N_2_ fixation, and potentially by 22% through nitrate/nitrite reduction to ammonium. Additional ammonium, isotopically not labelled and thus not accounted for in our analyses, might have originated from N_2_ fixation prior to our sampling. Ammonium uptake was presumably facilitated by ammonium concentrations within colonies that exceeded the half-saturation constant for ammonium uptake in N limited plankton (*K*_m_ ~ 0.05–0.25 µM) [71, 72]. *Trichodesmium* can indeed fix N_2_ and utilise combined N simultaneously when grown in culture [64]. Likewise, only half of the *Trichodesmium* cells have been shown to fix N_2_ in nature [73], and newly fixed N could be tracked from *Trichodesmium* to associated heterotrophic bacteria in the same colonies as studied herein (data shown in Fig. 4 and Table 4 in ref. [23]). Extracellular ammonium release may thus be an important transfer mechanism of newly fixed N between N_2_-fixing and non-N_2_-fixing *Trichodesmium* cells [74, 75], as conceptualised earlier [64].

Organic compounds add another level of complexity to the N cycling network in *Trichodesmium* colonies, which we did not account for in our incubations. In the Caribbean Sea and Atlantic Ocean, *Trichodesmium* has been shown to release up to half of the recently fixed N_2_ as DON [29]. The release consisted primarily of dissolved free amino acids, with release rates of 100 pmol glutamate colony^−1^ h^−1^ [27]. Such release rates could have compensated for the herein reported N deficit. Amino acids and even carbohydrates are indeed effectively assimilated within field-sampled *Trichodesmium* colonies; yet, it remains uncertain whether they are preferably assimilated by *Trichodesmium* or their epibionts [71, 76].

Nitrate consumption could have potentially fuelled the apparent N deficit during our nitrate-enriched incubations. However, actual nitrate availability is commonly low for *Trichodesmium* and other associated autotrophs under natural conditions. Our simulations predicted nitrate depletion in the colony centre, even after we added 1 µM of nitrate (Figs. 3d and 4), which exceeded the commonly prevailing nitrate concentrations at station ALOHA [77]. Thus, nitrate availability was diffusion-limited in *Trichodesmium* colonies. The ability of *Trichodesmium* consortia to instantaneously utilise nitrate when available may become quantitatively important during nutrient entrainment events or when colonies enter nitrate-enriched water layers. For instance, micromolar-levels are found close to the nitracline, at which *Trichodesmium* can occasionally be present in the sampling area [78, 79].

About 10% of the consumed nitrate was recovered as ammonium. Whether nitrate was reduced to ammonium via assimilatory (N incorporation) or dissimilatory pathways (N transformation coupled to energy production) cannot be determined from our experimental design. The genetic potential is present for both. Assimilatory nitrate reduction is directly encoded in *Trichodesmium*, and dissimilatory nitrate reduction in their associated epibionts [15, 38]. Still, assimilation seems more likely since oxygen concentrations of at least 50% air-saturation argue against anaerobic dissimilatory nitrate reduction to ammonium. Nitrite reduction rates were consistently lower than nitrate reduction to nitrite and/or ammonium, indicating that nitrate was preferentially assimilated over nitrite and subsequently recycled. It further indicates a potential niche separation of different taxa. For instance, transcripts for nitrate reductase (*narG*) were demonstrated to be associated with Bacteroides, whereas those for nitrite reductase (*nirB*) were ascribed to Gammaproteobacteria in *Trichodesmium* cultures [16].

### Nitrification and denitrification

Nitrification was insignificant although oxygen was plentiful even during darkness, and ammonium concentrations that were predicted to be high within colonies (Figs. 3c, d and 4). Consistently, nitrification genes were not found in *Trichodesmium* colonies that were sampled at Station ALOHA a few months prior to our sampling [15]. Furthermore, nitrifying archaea, as abundant nitrifiers in the marine environment [80], could not be detected in natural colonies in other oceanic regions [32, 33]. Nitrification also seems negligible in other aggregates, including N_2_-fixing colonies of *Nodularia* [22] and decaying diatoms [21]. However, nitrification genes have been found in *Nodularia* colonies [81], and nitrifying microbes could be detected in sinking particles [82]. The rather slow growth rates and light sensitivity of nitrifying microbes [83, 84] are likely to restrict their thriving in phytoplankton colonies. Moreover, ammonium and nitrite oxidation yields less energy compared to oxic respiration [19], and phytoplankton are considered to outcompete nitrifiers, in terms of ammonium utilisation, under nitrate-replete conditions [85].

Even though net nitrification was insignificant in the present and most previous studies on phytoplankton colonies and aggregates, we cannot rule out that the potentially high nitrate consumption had masked any ^15^N-nitrate production during our ^15^N-ammonium incubations. We actually found an indication for partial nitrification in N_2_O production after ^15^N-ammonium incubations (Fig. 2, Table 3). These production rates were significantly different from controls but close to our estimated detection limit. Significant amounts of N_2_O were also produced during ^15^NO_3_^–^ incubations when nitrification was inhibited. Hypothetically, denitrifiers may have emitted substantially more N_2_O in the presence of oxygen compared to anoxic conditions [86], while the N_2_O consumption may have been linked to nitrification in an yet unknown manner. Robust conclusions on N_2_O cycling should thus await further investigations.

Complete denitrification was not detectable, presumably due to the low nitrate availability and high oxygen concentrations (≥50% air-saturation or ≥100 µM) even during darkness. Denitrifiers and denitrification-relevant gene expression (*nosZ*) have been shown to be present in natural *Trichodesmium* colonies [14, 87]. However, most denitrifiers are facultative aerobes/anaerobes, depending on oxygen and nitrate availabilities [88]. We also could not detect any anammox (anaerobic ammonium oxidation) since our ^15^N-nitrite incubations in combination with the measured ammonium release did not yield any significant ^29^N_2_ production. Presumably this was due to the generally slow growth rates of anammox bacteria [84], similar to nitrifiers; and oxygen concentrations well above zero in the herein studied colonies. In fact, oxygen concentrations rarely drop below 50% air-saturation in highly porous colonies and aggregates such as: *Trichodesmium* colonies ([25, 35, 58], this study), other phytoplankton colonies [89] or marine snow [21, 90–92]. In contrast, anoxia is only expected in large (≥1 mm) phytoplankton colonies and aggregates [22, 93]. Recently, anoxia has been detected in colonies formed in *Trichodesmium* cultures [58] but, to our knowledge, the lowest oxygen concentration measured in natural *Trichodesmium* colonies was 10% air-saturation (~20 µM) [24]. Given the thresholds for anaerobic denitrification and anammox of ≤20 µM in marine waters [94–98], complete denitrification and anammox may be a rare exception, and oxygen may be the energetically preferred electron acceptor over nitrate in natural *Trichodesmium* colonies.

We evaluated a long-standing aspect of *Trichodesmium* ecology by quantitatively exploring the potential microbial N cycling network in millimetre-sized colonies. The observed N cycling in puff-type colonies was imbalanced, with a high potential of gain and recycling processes but a low potential for loss by denitrification (Fig. 2). Thus, *Trichodesmium* cells (and its epibionts) appeared to act in concert to preserve new N from N_2_ fixation in close proximity, to fully benefit from bioavailable N in the oligotrophic near-surface ocean. *Trichodesmium* colonies may thus be described as consortia, reflecting associations of synergistic, mutualistic or syntrophic lifestyles [99, 100], in which the growth and elemental cycling are more efficient than on a single-population level.

## Supplementary information

Supplementary Information
