# Supplementary material for: Distinct nitrogen cycling and steep chemical gradients in Trichodesmium colonies
Source: ISME J. 2019 Oct 21;14(2):399–412. doi: 10.1038/s41396-019-0514-9 (PMC6976679; doi:10.1038/s41396-019-0514-9)
Supplement: Supplementary file 1 — Supplementary Information [file 41396_2019_514_MOESM1_ESM.docx]

# Supplementary Information

**Distinct nitrogen cycling and steep chemical gradients in *Trichodesmium* colonies**

Isabell Klawonn, Meri J Eichner, Samuel T Wilson, Nasrollah Moradi, Bo Thamdrup, Steffen Kümmel, Matthias Gehre, Arzhang Khalili, Hans-Peter Grossart, David M Karl, Helle Ploug

## Table S1. Environmental data collected from the Hawai'i Ocean Time-series program at station ALOHA. Reported data were assessed under <http://hahana.soest.hawaii.edu/hot/hot-dogs/cextraction.html> for surface water (ca. 0–10 m) sampled at different times of the day during September 14–16^th^, 2014.

| **Parameter (unit)** | **Value** |
| --- | --- |
| Mixed layer depth (m) | 50 |
| Dissolved inorganic carbon (µmol L^-1^) | 1937 |
| PO_4_^3-^ (µmol L^-1^) | 0.14±0.01 (*n*=3) |
| NO_3_^-^ + NO_2_^-^ (µmol L^-1^) | 0.005 |
| Silica (µmol L^-1^) | 1.29±0.26 (*n*=3) |
| Particulate carbon (µmol L^-1^) | 2.8 |
| Particulate nitrogen (µmol L^-1^) | 0.4 |
| Chlorophyll (µg L^-1^) | 0.06±0.01 (*n*=4) |

## Text S1. Isotope-additions during incubations

After isotope additions, final concentrations of nitrate, nitrite and ammonium exceeded typical *in situ* concentrations at the sampling station, in order to overcome diffusion-limited solute transport into the colonies. Final concentrations were ~1 µM (0.9±0.3 µM, mean±sd, *n*=18). These concentrations were chosen to keep an appropriate balance between nutrient enrichment over natural *in situ* concentrations (usually <<1 µM) and sufficient concentrations to avoid diffusion-limitation transport of nutrients into the colony centre.

The diffusive flux *J* was calculated from Fick’s 1^st^ law:

$J=-D_{ɑ}\frac{dC}{dr}$ Eq. S1

, where *D_ɑ_* was the diffusion coefficient (see Table S2), *dC* the concentration difference between ambient water and the colony centre (here assumed to be 1 µM, since the final nutrient concentration was ~1 µM in the water, and a depletion down to zero was assumed for the centre), and *dr* the distance from the colony surface to the centre (*r_tot_* = 807 µm). Accordingly, concentrations of 1 µM were expected to support diffusive fluxes at the colony–water interface of approx. 0.8 nmol cm^-2^ h^-1^, which corresponded to ~70 pmol colony^-1^ h^-1^ for a colony with *r_tot_* = 807 µm and a surface area *A* = 0.08 cm^2^. Thus, our measured rates, as listed in Table 3 in the main document, were not diffusion but rather reaction-limited, except for residual nitrate consumption. For the latter process, rates of ~70 pmol colony^-1^ h^-1^ were predicted to lead to nitrate depletion in the colony centre, as also demonstrated by our computer simulations (Figure 4D).

Diffusive time scales can be estimated from *l^2^/D* with *l* as the distance, and *D* as diffusion coefficient. Thus, added nitrate or ammonium were estimated to diffuse from the ambient into the colony centre within minutes (3 min), given that the radius was ~800 µm (the diffusion coefficients are listed below in Table S2).

## Text S2. Set-up of GC-IRMS for stable-isotope analyses

The production of ^15^N-labeled N_2_ (mass 29 and 30) and N_2_O (measured as mass 29 and 30 after reduction to N_2_) were determined by headspace analysis using gas chromatography isotope-ratio mass spectrometry (GC-IRMS, concentration precision ±5%) on a DeltaV plus (Thermo Scientific) interfaced via a ConFlo III (Thermo Scientific) to a custom-built GC setup at the University of Southern Denmark in Odense [[1](#_ENREF_1)].

^15^N-nitrate, ^15^N-nitrite and ^15^N-ammonium were converted to N_2_ gas and also analysed via GC-IRMS at the UFZ in Leipzig, Germany (concentration precision ±5%). An analytical train, featuring a GC unit (7890A, Agilent Technologies, Germany) and a GC IsoLink interface, including a combustion reactor (Thermo Fisher, Bremen, Germany, 1000°C), a reduction reactor containing a copper catalyst (IVA Analysentechnik, Meerbusch, Germany, 650°C) and a liquid N_2_ trap, was connected via a ConFlo IV open split system to a MAT 253 IRMS (Thermo Fisher, Bremen, Germany). Samples were separated isothermally at 40°C on a CP-PoraBond Q column (50 m x 0.32 mm, 5 µm film thickness, JW Scientific, Germany). ^15^N-ammonium was chemically converted to N_2_ with alkaline hypobromite [[2](#_ENREF_2)], ^15^N-nitrate was reduced to nitrite with cadmium and ^15^N-nitrite was reduced to N_2_ with sulfamic acid [[3](#_ENREF_3)]. Prior to the chemical treatments, the water was gently filtered (0.2 µm). The conversion efficiency was tested with ^15^N-nitrate, ^15^N-nitrite and ^15^N-ammonium standards in the expected concentration range of 0–500 nM and the obtained conversion factors (0.55–1.00) were applied. The experimental and analytical setups allowed detection of N-production rates per colony of approximately 0.05 pmol h^-1^.


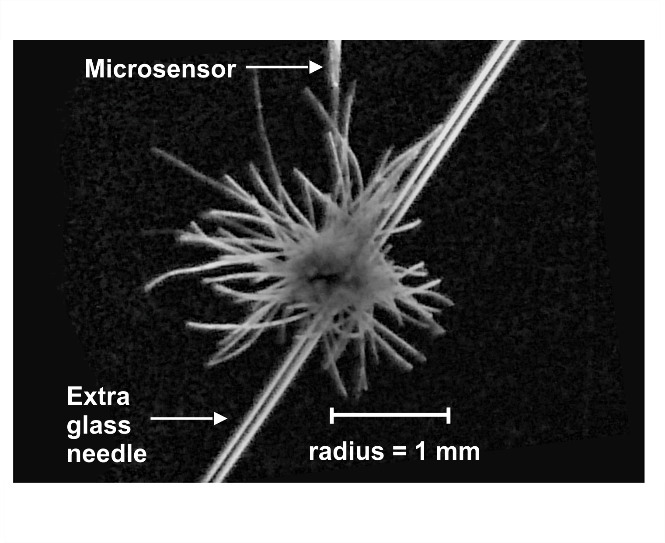


## Figure S1. Photograph of *Trichodesmium* colony. The extra needle was used for stabilizing the positive buoyant colony during microsensor measurements in water. The original photo was converted to a B/W image, with added black background and adjusted brightness, contrast and intensity.

## Text S3. Numerical modelling of solute gradients and concentration fields: Computational domain and boundary conditions

We used a recently developed mathematical model [[4](#_ENREF_4)] to simulate concentration fields of oxygen, ammonium and nitrate. As a novelty, the model extended the commonly used advection–diffusion equation with a reaction term for microbial activity. Physical mass transfer and biologically-mediated transformation processes of solutes are thus adequately represented in the model.

Colonies were modelled as porous spheres and trichomes as solid cylinders, both with axial symmetry along the *z*-axis (Figure 1 in the main document). Because of the geometrical symmetry, the 3D mathematical problem was reduced to a 2D problem; that is, Eq. 1 (in the main document) was solved in a 2D coordinate system, considering only the *z* and *ρ* coordinates [[5](#_ENREF_5)]. A deviation of Eq. 1 (main document), excluding the reaction term, has been presented earlier [[Eq. 3.24 in ref. 6](#_ENREF_6)]. To calculate the concentration field of each solute inside/around colonies and single trichomes, the equation

$\varepsilon\frac{dC_{\alpha}}{dt}=\frac{1}{\rho}\frac{\partial}{\partial\rho}\left( \varepsilon D_{\alpha}\rho\frac{\partial C_{\alpha}}{\partial\rho} \right)+\frac{\partial}{\partial z}\left( \varepsilon D_{\alpha}\frac{\partial C_{\alpha}}{\partial z} \right)+nR_{\alpha}\text{ }$ Eq. S2

was solved numerically using the lattice Boltzmann method, as described previously [[5](#_ENREF_5)] (see main document for parameter definitions, please). The lattice Boltzmann method is a computational method that was originally developed to solve flow equations on certain lattice-based grids and is suitable to deal with complex geometries, but it is also powerful to solve advection–diffusion–reaction equations at microscopic/mesoscopic levels [[7](#_ENREF_7), [8](#_ENREF_8)]. It hereby describes the temporal dynamics of molecule populations (at each lattice node) that move along a set of discrete directions to the neighbouring nodes, collide with the populations coming from other nodes and then propagate around accordingly to the neighbouring nodes (Figure S2A). The computational domain was mapped by the so called D2Q9 lattice. The associated lattice Boltzmann version of Eq. S2, accounting for the symmetry along the z-axis, was solved at each lattice node in the entire computational domain [[5](#_ENREF_5), [9](#_ENREF_9)]. All simulation results are shown for steady-state conditions, i.e., the systems evolved in time until the temporal change in the concentration field became practically zero (*dC_α_ / dt = 0*).

The D2Q9 lattice-based grid included consecutive refinement levels (Figure S2B). To resolve high spatial resolutions, the simulations were performed on a large lattice size of 513 x 513 nodes with hierarchies of five nested girds for the colony, and four nested grids for the single trichome. The centre of the cross section of the colony and single trichome were set at the origin of the *ρ–z* coordinate system. The colony radius *r_tot_* was set to 180, and the height *H* and length *L* of the trichome to 370 and 4 lattice nodes, respectively. The spatial resolution was circa 4.5 μm for the colony and 1.7 µm for the trichome.

Theoretically, the colony (or single trichome) was assumed to be in an unbounded region. Practically, this imposes a far-field boundary condition, that is, the solute concentration at a distance sufficiently far away from the colony/trichome should equal the solute concentration in the ambient seawater (Dirichlet boundary condition). The grid refinement technique allowed for imposing the boundary condition at a distance sufficiently far away from the colony/single trichome, i.e., at the outer boarder of the coarsest grid (Figure S2B). For the colony, the boundary conditions were imposed at a distance of 26× *r_tot_*. For the trichome, the boundary conditions were imposed at a distance of ca 12× *H*/2 along the z-axis, and 1024× *L*/2 along the *ρ*-axis. Since there was no advection in the system, no ad-hoc boundary condition was imposed at the surface of the colony/trichome. However, the nodes in the modelled colony/trichome were conditioned by reaction, while the reaction rate was set to zero on nodes in the ambient water.

The solute concentration was initially (at *t*=0) set to the ambient solute concentration, *C_0(α)_*, in the entire computational domain. Since the total volume of cells in the colony accounted for <0.1% of the total colony volume, cells were considered as solid objects from which solutes were released or consumed, without considering their internal solute concentrations. The typical simulation (CPU) time for each colony to reach steady-state was 3–4 weeks on a standard CPU with a single core, as the mass transport by diffusion is a slow process over large distances (diffusion time scales are in the range of *l^2^/D* with *l* as the distance, and *D* as diffusion coefficient). The computational time, however, strongly depended on the initial condition (i.e. the applied concentration at time zero), and thus, setting the initial concentration closer the steady-state solution could significantly reduce the computational time. Further equations and explanations are presented in the initial model description [[4](#_ENREF_4), [5](#_ENREF_5)].


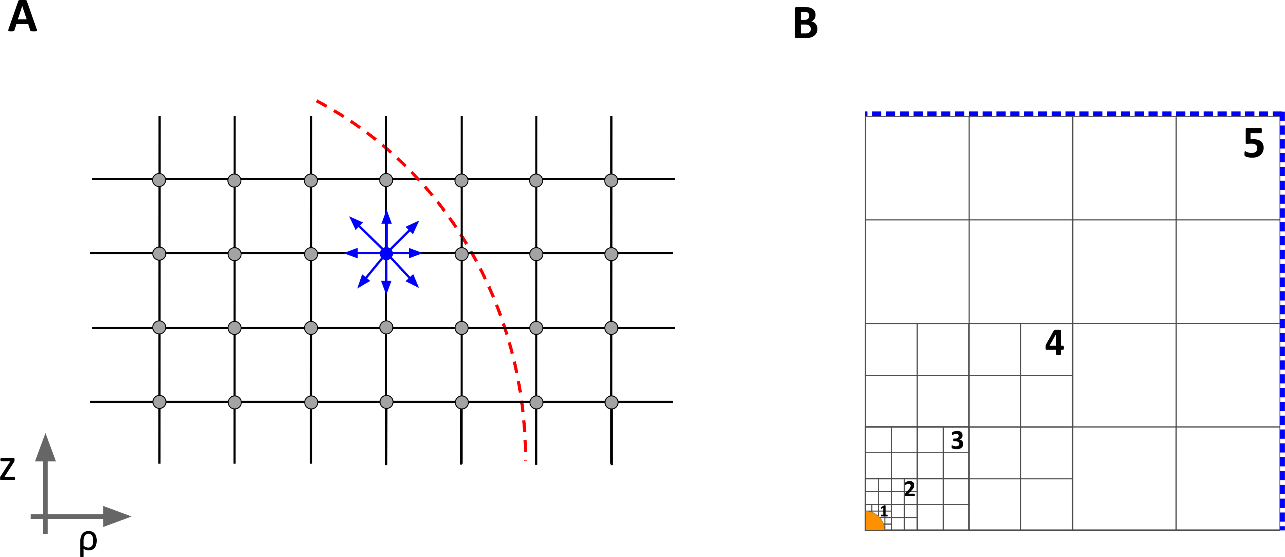


## Figure S2. (A) Scheme of the D2Q9 lattice used as the underlying grid for the computational domain. The blue arrows show the eight discrete directions along which molecule populations can travel to the neighbouring nodes, collide and propagate at each time step. This dynamic behaviour took place simultaneously at all lattice nodes in the computational domain. The dashed red line illustrates the interface of the modelled colony and the ambient water. (B) Simplified scheme of a grid refinement technique (of level 5) for the simulation of consumption/release of solutes in a *Trichodesmium* colony. For clarity, only one quarter of the entire domain is shown. The spatial resolution of the coarsest grid was 16-times larger than that of the finest grid. The small orange circle in the finest grid shows the position of the modelled colony in the computational domain. The blue dashed lines show the outer boarder of the coarsest grid at which the boundary conditions were imposed.

## Table S2. Input parameters used for computer simulations. The maximum reaction rates *V_m_*_(_*_ɑ_*_)_ were obtained by normalizing colony-specific activity rates to single-cell units (by dividing the highest average rate listed in Table 3 by 6,000 *Trichodesmium* cells). Rates of total nitrate consumption represent the sum of nitrate reduction to ammonium/N_2_ plus the residual nitrate consumption. Diffusion coefficients were extracted from [[10](#_ENREF_10), [11](#_ENREF_11)] and adjusted to 25°C. N/A – not applicable.

|  | **Oxygen respiration (dark)** | **Oxygen production (light)** | **Nitrate reduction to ammonium (average day/night)** | **Total nitrate consumption (average day/night)** | **Ammonium release (during N_2_-fixation, day)** |
| --- | --- | --- | --- | --- | --- |
| Maximum reaction rate *V_m(ɑ)_* (fmol cell unit^-1^ h^-1^) | 292 | 478 | 1.8 | 13.1 | 3.2 |
| Diffusion coefficient *D_(ɑ)_* (cm^2^ s^-1^) | 2.24E-05 | 2.24E-05 | 1.81E-05 | 1.81E-05 | 1.89E-05 |
| Ambient concentration *C_0(ɑ)_* (µM) | 212.5 | 212.5 | 1.0 | 1.0 | 0.2 |
| Half-saturation constant *K_m(ɑ)_* (µM) | 1.0 | N/A | 20 | 20 | N/A |

## References

1. Dalsgaard T, Thamdrup B, Farías L, & Revsbech NP (2012) Anammox and denitrification in the oxygen minimum zone of the eastern South Pacific. *Limnol. Oceanogr.* 57(5):1331-1346.

2. Warembourg FR (1993) Nitrogen fixation in soil and plant systems. *Nitrogen Isotope Techniques*, ed Blackburn RKH (Academic Press, San Diego), pp 127-156.

3. Füssel J, Lam P, Lavik G, Jensen MM, Holtappels M, Günter M *et al.* (2012) Nitrite oxidation in the Namibian oxygen minimum zone. *ISME J.* 6(6):1200-1209.

4. Moradi N, Liu B, Iversen M, Kuypers MMM, Ploug H, & Khalili A (2018) A new mathematical model to explore microbial processes and their constraints in phytoplankton colonies and sinking marine aggregates. *‎Sci. Adv.* 4(10):eaat1991.

5. Liu B, Kindler K, & Khalili A (2012) Dynamic solute release from marine aggregates. *Limnol. Oceanogr. Fluid Environ.* 2(1):109-120.

6. Nield DA & Bejan A (2017) Chapter 3 - Mass Transfer in a Porous Medium: Multicomponent and Multiphase Flows. *Convection in Porous Media*, eds Nield DA & Bejan A (Springer Nature, Cham, Switzerland), 5 Ed, pp 57-84.

7. Succi S, Moradi N, Greiner A, & Melchionna S (2014) Lattice Boltzmann modeling of water-like fluids. *Frontiers in Physics* 2(22):1-14.

8. Succi S (2001) *The Lattice Boltzmann equation for fluid dynamics and beyond* (Clarendon Press, Oxford) p 308.

9. Ma Q, Chen Z, & Liu H (2017) Multiple-relaxation-time lattice Boltzmann simulation for flow, mass transfer, and adsorption in porous media. *Physical Review E* 96(1):013313.

10. Ramsing N & Gundersen J Seawater and Gases. Unisense A/S, Denmark.

11. Li YH & Gregory S (1974) Diffusion of ions in sea water and in deep-sea sediments. *Geochim. Cosmochim. Acta* 38(5):703-714.
